# Effect of Platelet-Rich Plasma on M1/M2 Macrophage Polarization

**DOI:** 10.3390/ijms22052336

**Published:** 2021-02-26

**Authors:** Ryoka Uchiyama, Eriko Toyoda, Miki Maehara, Shiho Wasai, Haruka Omura, Masahiko Watanabe, Masato Sato

**Affiliations:** 1 Department of Orthopaedic Surgery, Surgical Science, Tokai University School of Medicine, 143 Shimokasuya, Isehara, Kanagawa 259-1193, Japan; 9bmrm002@mail.u-tokai.ac.jp (R.U.); etoyoda@tokai-u.jp (E.T.); m-maehara@tsc.u-tokai.ac.jp (M.M.); s4.1x2.5s@gmail.com (S.W.); hrk.omr@gmail.com (H.O.); masahiko@is.icc.u-tokai.ac.jp (M.W.); 2 Center for Musculoskeletal innovative Research and Advancement (C-MiRA), Tokai University Graduate School of Medicine, 143 Shimokasuya, Isehara, Kanagawa 259-1193, Japan

**Keywords:** platelet rich plasma, autologous protein solution, macrophage

## Abstract

Osteoarthritis of the knee (OAK) is a chronic degenerative disease and progresses with an imbalance of cytokines and macrophages in the joint. Studies regarding the use of platelet-rich plasma (PRP) as a point-of-care treatment for OAK have reported on its effect on tissue repair and suppression of inflammation but few have reported on its effect on macrophages and macrophage polarization. Based on our clinical experience with two types of PRP kits Cellaid Serum Collection Set P type kit (leukocyte-poor-PRP) and an Autologous Protein Solution kit (APS leukocyte-rich-PRP), we investigated the concentrations of humoral factors in PRPs prepared from the two kits and the effect of humoral factors on macrophage phenotypes. We found that the concentrations of cell components and humoral factors differed between PRPs purified using the two kits; APS had a higher concentration of M1 and M2 macrophage related factors. The addition of PRP supernatants to the culture media of monocyte-derived macrophages and M1 polarized macrophages revealed that PRPs suppressed M1 macrophage polarization and promoted M2 macrophage polarization. This research is the first to report the effect of PRPs purified using commercial kits on macrophage polarization.

## 1. Introduction

Osteoarthritis of the knee (OAK) is a chronic degenerative disease that damages articular cartilage. OAK is characterized by aberrant cartilage metabolism, osteophyte formation, synovial tissue thickening, and macrophage infiltration. These changes contribute to the progress of OAK, resulting in joint pain and dysfunction [1,2]. There are about 25 million patients with OAK in Japan, and the number is expected to increase in tandem with Japan’s hyper-aging population [3]. No treatment currently exists that lead to a complete recovery from OAK. Conservative treatments (i.e., exercise, oral treatment, intra-articular injection of hyaluronic acids) are performed for mild cases, and surgical treatments (i.e., arthroscopic debridement of the knee joint, high tibial osteotomy, and total knee arthroplasty) for severe cases [4,5]. Joint treatment with platelet-rich plasma (PRP) has recently attracted attention as a point-of-care treatment option to bridge the gap between conservative and surgical treatments.

PRP is an autologous blood product generated by centrifuging whole blood and concentrating platelets, containing diverse growth factors and cytokines [6]. The high concentration of anti-inflammatory factors in PRPs may suppress inflammation, growth factors may promote tissue repair, and in total these factors may improve symptoms [7,8]. Efficacy of platelet concentrates in promoting wound healing and tissue regeneration has been at the center of scientific debate over the past few decades [9]. Various purification kits have recently been marketed as this treatment has become more widespread. However, the blood cell components and humoral factors in PRPs depend on the purification method. Leukocyte-poor PRP (LP-PRP) lacks leukocytes whereas leukocyte-rich PRP (LR-PRP) do contain leukocytes. We have clinical experience with both types of kits: Cellaid Serum Collection Set P type kit (LP-PRP) and an Autologous Protein Solution kit (APS, LR-PRP). APS, with an added dehydration step using polyacrylamide beads during purification, has been reported to achieve a high concentration of not only platelets and leukocytes but also humoral factors such as IGF-1 [6]. However, there is no consensus on how differences in the components of PRP products affect their therapeutic efficacies.

One possibility is the effect of PRPs on macrophages. Macrophages are immune cells that play a crucial role in innate immunity and also participate in tissue repair and remodeling [10]. Macrophages can be polarized into two phenotypes in response to stimuli from their microenvironment. Classically activated macrophages (M1 macrophages) are induced by, for example, interferon-gamma (IFN-γ), tumor necrosis factor-alpha (TNF-α), and bacterial-derived lipopolysaccharides (LPS), and are known to release inflammatory cytokines that promote tissue damage and inflammation [11,12,13]. Alternatively activated macrophages (M2 macrophages) are induced by, for example, interleukin-4 (IL-4) and IL-13, and are known to release anti-inflammatory cytokines that promote tissue remodeling and suppress inflammation [14,15].

Recent studies have shown that M1 macrophages are present in the synovium and synovial fluid in OAK patients and involved in disease progression, suggesting they may be targeted for treatment [16,17,18,19]. Many studies have reported PRPs suppressing inflammation and inducing articular cartilage repair, but few studies have reported the effect of PRPs on macrophage phenotypes [20,21,22].

In this study, LP-PRP and APS were purified from peripheral blood of healthy subjects using two PRP preparation kits in which we have clinical experience. We then compared the concentrations of humoral factors related to M1/M2 macrophage polarization in the respective PRPs, and then added the respective PRP supernatants to the culture media of monocyte-derived macrophages (MDMs) to investigate their effect on macrophage phenotypes. 

## 2. Results

### 2.1. Hematological Analysis of PRPs

**Figure 1:**
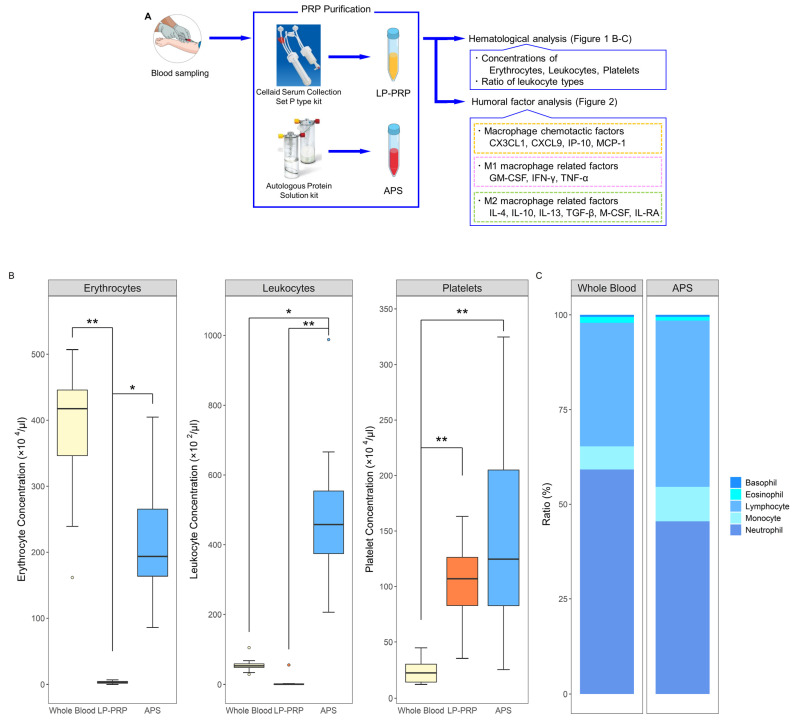
Purification and analyses of platelet-rich plasmas (PRPs) from two kits. (**A**) Flow of PRP purification and analyses. From each of the 12 healthy subjects, 120 mL of peripheral blood was collected and was added to 12 mL of anticoagulant citrate-dextrose solution. Leukocyte-poor PRP (LP-PRP) and Autologous Protein Solution (APS) were purified using their respective kits, and hematological analysis was performed immediately following purification. Humoral factor analysis was performed with frozen stored samples for various macrophage related factors. (**B**) Comparison of leukocyte, erythrocyte, and platelet concentrations in LP-PRP and APS. (**C**) Comparison of the ratios of leukocyte types in whole blood and APS. *n* = 12 PRP donors for each kit. * *p* < 0.05, ** *p* < 0.01.

From each healthy donor, 120 mL of peripheral blood was collected and 60 mL per kit was used to prepare both LP-PRP and APS. Immediately after preparation, hematological analysis was performed for whole blood, LP-PRP, and APS (Figure 1A). Erythrocyte concentration was negligible for LP-PRP while it was lower for APS compared to whole blood. Leukocyte concentration was negligible for LP-PRP while it was higher for APS compared to whole blood. Platelet concentrations were higher for both LP-PRP and APS compared to whole blood and did not significantly differ between LP-PRP and APS (Figure 1B). A comparison of the ratio of leukocyte types contained in whole blood and APS revealed that in APS, the ratios of neutrophils and eosinophils were higher while the ratios of monocytes and lymphocytes were lower (Figure 1C). According to the coding system presented by Kon et. al., the PRP codes for LP-PRP and APS are 210-00-00 and 214-15-10, respectively [23].

### 2.2. Analysis of Humoral Factors

**Figure 2:**
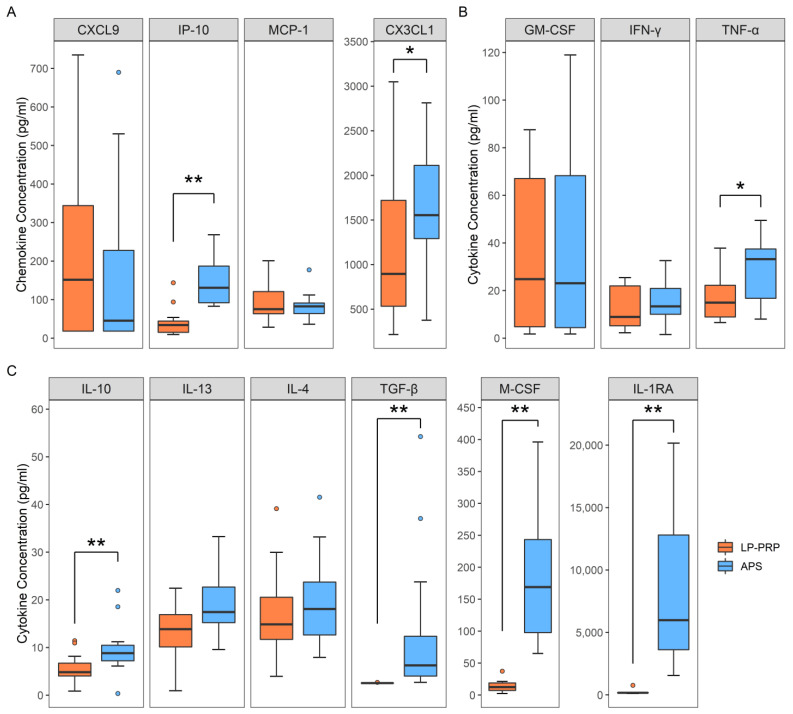
Analysis of humoral factors in PRPs. Comparison of concentrations of each factor in LP-PRP and APS. (**A**) Macrophage-recruitment factors (**B**) M1 macrophage related factors (**C**) M2 macrophage related factors. *n* = 12 PRP donors for each kit. * *p* < 0.05, ** *p* < 0.01.

We investigated the effect of PRPs on macrophage polarization by analyzing the humoral factors in the respective PRPs. Macrophages respond to factors such as C-X-C motif chemokine ligand 9 (CXCL9), interferon gamma-induced protein 10 (IP-10), C-XXX-C motif chemokine ligand 1 (CX3CL1), and macrophage chemoattractant protein 1 (MCP-1), and are thereby recruited to areas of inflammation. M1 macrophages respond to and are polarized by granulocyte macrophage colony-stimulating factor (GM-CSF), TNF-α, IFN-γ, while M2 macrophages respond to and are polarized by macrophage colony-stimulating factor (M-CSF), IL-4, IL-10, IL-13, IL-1 receptor antagonist (IL-1RA), and transforming growth factor-beta (TGF-β) [14]. As a result of quantitative analysis, both LP-PRP and APS contained all of these factors. A comparison of the concentrations of these factors in LP-PRP and APS showed that the concentration of macrophage-recruitment chemokine IP-10 and CX3CL1 was significantly higher in APS (Figure 2A). Moreover, the concentrations of the M1 macrophage related factor TNF-α (Figure 2B) and the M2 macrophage related factors M-CSF, IL-10, IL-1RA, and TGF-β (Figure 2C) were also significantly higher in APS. These results showed that LP-PRP and APS differ in the concentrations of cytokines involved in macrophage polarization with higher concentrations of both M1 and M2 macrophage related factors in APS.

### 2.3. Effect of the Addition of PRP Supernatants on Macrophage Phenotypes

**Figure 3:**
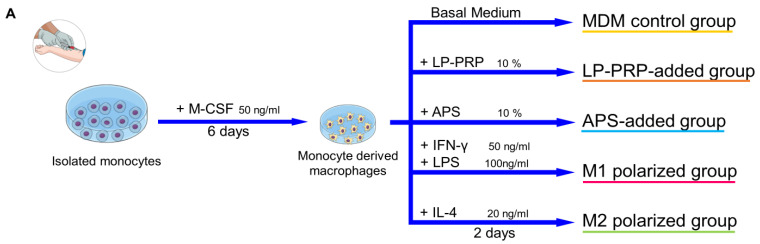

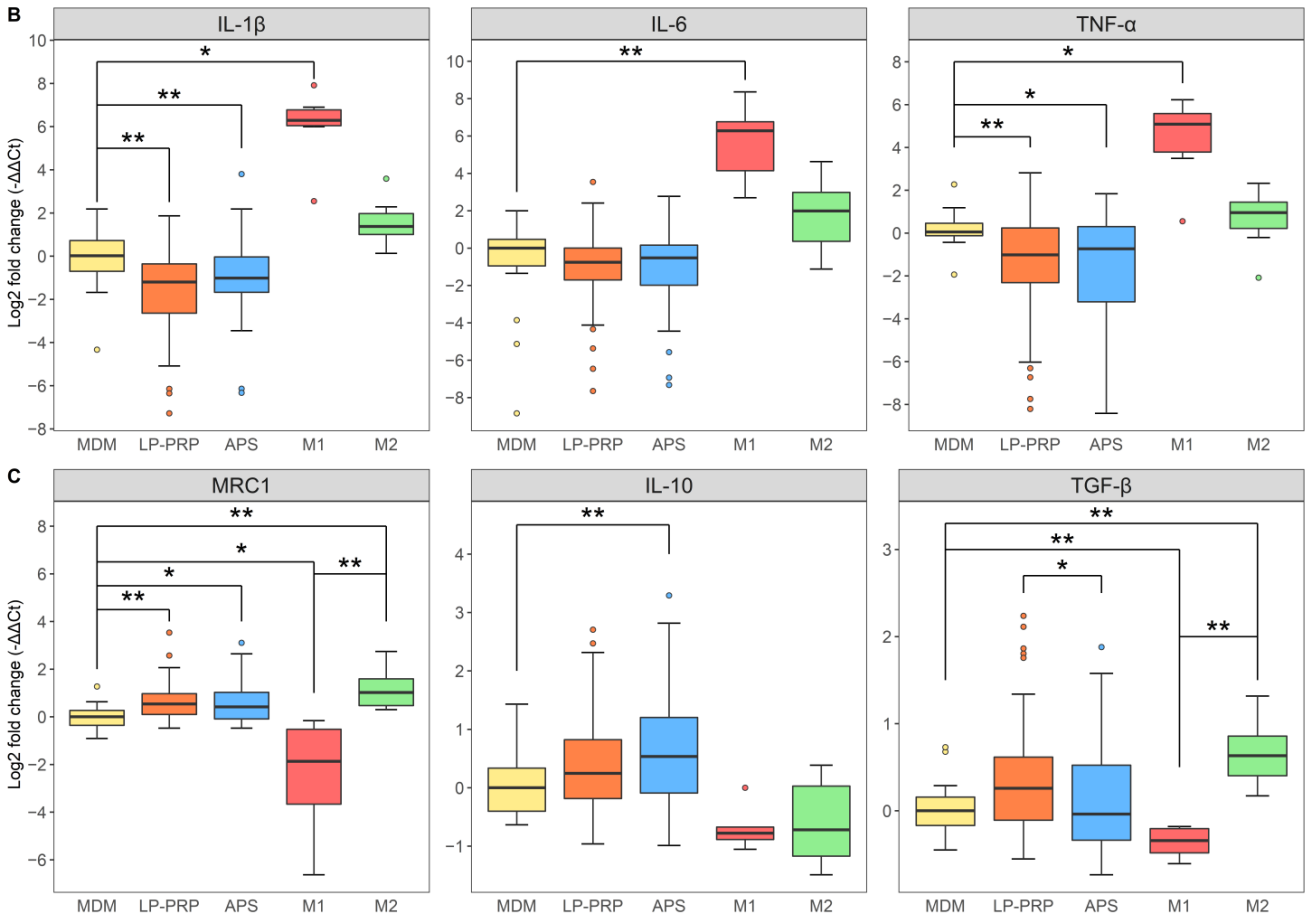
Effect of PRP supernatants on gene expression of M1/M2 macrophage markers. (**A**) Summary of this experiment. CD14 + monocytes were isolated from peripheral blood using density gradient centrifugation and magnetic beads. Monocytes were cultured in basal medium supplemented with 10% FBS containing M-CSF at 37 °C under 5% CO_2_ for six days, then the medium was replaced by fresh basal medium supplemented with 10% FBS containing supernatants obtained from LP-PRP or APS, and the cells were cultured for another two days. (**B**) Gene expression of M1 macrophage markers (IL-1β, IL-6, TNF-α) and (**C**) M2 macrophage markers (MRC1, IL-10, TGF-β). Data were analyzed through qRT-PCR. -ΔΔCt values were calculated using GAPDH as an internal control. Monocyte-derived macrophages (MDM) control group served as negative control, and M1 and M2 polarized groups served as positive controls. MDM control group: 6 monocyte donors, 5 experiments, total *n* = 30; LP-PRP- and APS-added groups: 6 monocyte donors, 12 PRP donors, *n* = 72 group; M1 and M2 polarized groups: 6 monocyte donors, 2 or 3 experiments, *n* = 14 per group. * *p* < 0.05, ** *p* < 0.01.

Next, we investigated whether PRPs affect macrophage phenotypes and whether the effect differs depending on the purification kit. M1 macrophages express IL-1β, IL-6, and TNF-α, while M2 macrophages express MRC1, IL-10, and TGF-β [14]. Monocytes were isolated from human peripheral blood and cultured in M-CSF for six days, then supernatants from each PRP was added to the culture media, and the cells were cultured for another two days. Then, the effect of PRP supernatants on the expression of the M1/M2 macrophage marker genes was confirmed by quantitative reverse transcription-polymerase chain reaction (qRT-PCR) (Figure 3A). MDM control group served as negative control, and M1 and M2 polarized groups served as positive controls. The expression of IL-1β and TNF-α, which are M1 macrophage markers, was lower in the LP-PRP- and APS-added groups compared with the MDM control group, and the expression of IL-6 also tended to be lower. However, there was no significant difference in gene expression between the addition of PRP supernatants purified using the two kits (Figure 3B). The expression of MRC1, which is an M2 macrophage marker, was higher in the LP-PRP- and APS-added groups compared with the MDM control group. The expression of IL-10 was higher in the APS-added group compared with the MDM control group. The expression of TGF-β was higher in the LP-PRP-added group compared with the APS-added group, but no difference was detected between LP-PRP and APS-added groups compared with the MDM control group (Figure 3C).

**Figure 4:**
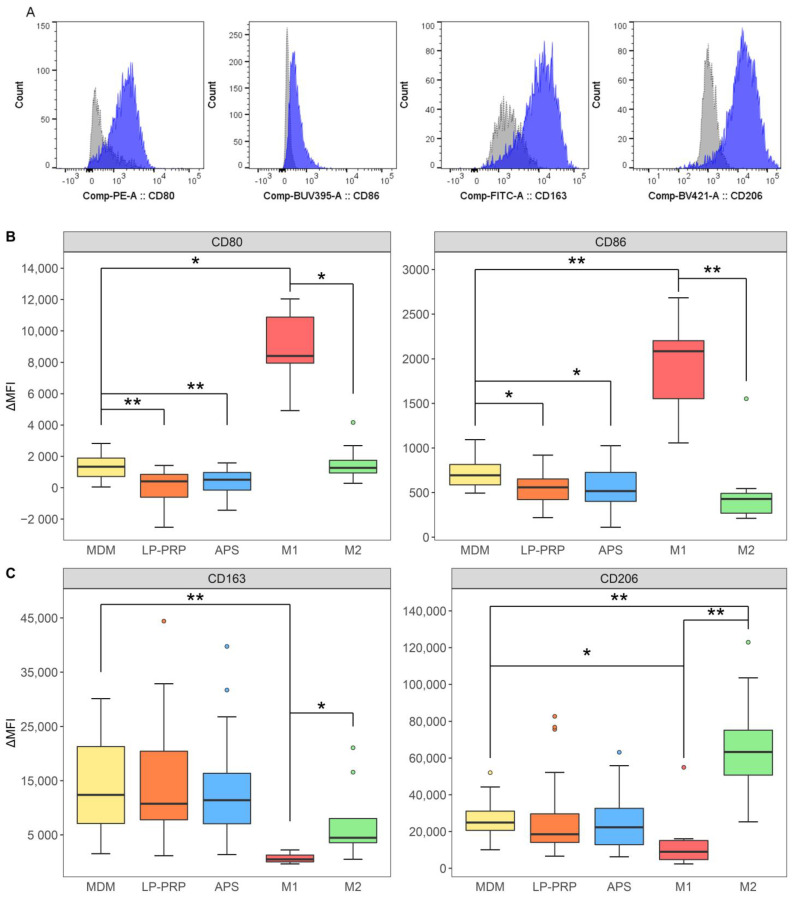
Flow cytometric analysis of M1/M2 macrophage cell surface markers. Cells were cultured under the same protocol as Figure 3. (**A**) A histogram representation of typical flow cytometry results. Gray and dashed lines: isotype control; blue and solid lines: signals for each antibody. Mean fluorescence intensity (MFI) values of each antibody were used to calculate ΔMFI values: ΔMFI = MFI Sample—MFI Isotype. (**B**) M1 macrophage markers. (**C**) M2 macrophage markers. MDM control group served as negative control, and M1 and M2 polarized groups served as positive controls. MDM control group: 6 monocyte donors, 5 experiments, *n* = 30; LP-PRP- and APS-added groups: 6 monocyte donors, 12 PRP donors, *n* = 72 per group; M1 and M2 polarized groups: 6 monocyte donors, 2 or 3 experiments, *n* = 14 per group. * *p* < 0.05, ** *p* < 0.01.

Similar verification experiments were performed for cell surface markers using flow cytometry. M1 macrophages express CD80 and CD86, while M2 macrophages express CD163 and CD206 on the cell surface [14]. A typical histogram is shown in Figure 4A. The values were calculated and compared based on difference of the mean fluorescence intensities between the antibody and isotype control. The expression of CD80 and CD86, which are M1 macrophage markers, decreased in the LP-PRP- and APS-added groups compared with the MDM group, but no difference was observed between the purification kits (Figure 4B). On the other hand, there was no difference in the expression of the M2 macrophage markers CD163 and CD206 between the MDM control group and the LP-PRP- or APS-added groups (Figure 4C).

These results showed that the addition of PRP supernatants decreased the expression of M1 macrophage markers, but there was no difference between the purification kits. On the other hand, the expression of M2 macrophage surface markers tended to be maintained or increased by the addition of PRP supernatants while the gene expression of IL-10 increased in the APS-added group and TGF-β increased in the LP-PRP-added group.

### 2.4. Effect of PRPs on M1 Macrophages

**Figure 5:**
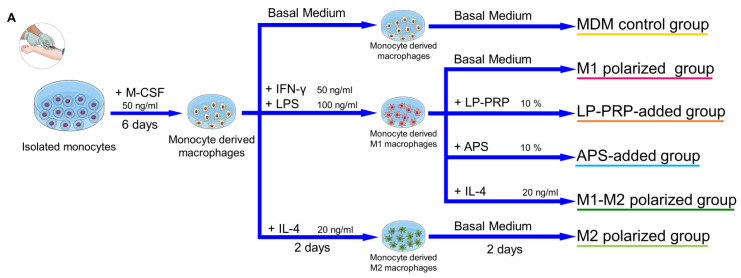

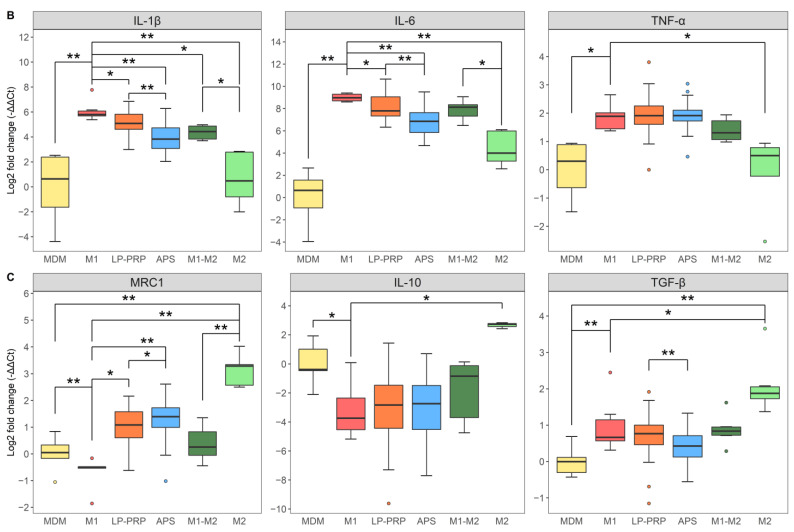
Effect of PRPs on M1 macrophages. (**A**) After CD14 + monocytes were isolated by the same method as described in Figure 3, they were cultured in a basal medium supplemented with 10% FBS containing M-CSF at 37 °C under 5% CO_2_. After six days, the media was replaced by fresh basal medium supplemented with 10% FBS containing IFN-γ + LPS, and the cells were cultured for another two days to polarize them to M1 macrophages. The medium was removed, the basal medium supplemented with 10% FBS containing supernatants obtained from LP-PRP or APS was added, and the cells were cultured for another two days. (**B**) Expression of M1 macrophage markers (IL-1β, IL-6, TNF-α). (**C**) Expression of M2 macrophage markers (MRC1, IL-10, TGF-β). Data were analyzed through qRT-PCR. -ΔΔCt values were calculated using GAPDH as an internal control. MDM control and M1 polarized groups served as negative controls; M1-M2 and M2 polarized groups served as positive controls. MDM control, M1 polarized, M1-M2 polarized, M2 polarized groups: 6 monocyte donors, 1 experiment, *n* = 6 per group; LP-PRP- and APS-added groups: 6 monocyte donors, 12 PRP donors, *n* = 72 per group. * *p* <0.05, ** *p* <0.01.

M1/M2 macrophages can change their phenotypes once polarized [24], and, thus, we investigated whether PRPs can induce the polarization of M1 macrophages to M2 macrophages. Monocytes were isolated from human peripheral blood and cultured in M-CSF for six days. MDMs were polarized to M1 macrophages and cultured for two days, then each PRP was added to the culture medium, and the cells were cultured for another two days (Figure 5A). MDM control and M1 polarized groups served as negative controls; M1-M2 and M2 polarized groups served as positive controls. The expressions of IL-1β and IL-6, which are M1 macrophage markers, decreased in the LP-PRP- and APS-added groups, and suppression was greater in the APS-added group than in the LP-PRP-added group (Figure 5B). Expression of the M2 macrophage marker MRC1 increased by the addition of PRPs and this increase was greater in the APS-added group than in the LP-PRP-added group. The expression of TGF-β was higher in the LP-PRP-added group than in the APS-added group (Figure 5C). These results showed that PRPs can repolarize M1 macrophages to M2 macrophages.

## 3. Discussion

Platelets play an important role in the process of hemostasis and tissue repair by gathering at injury sites and secreting various cytokines and growth factors to initiate the repair process [25]. The use of PRPs is based on the hypothesis that they eliminate the imbalance of cytokines in joints following the injection of large amounts of platelets, which release anti-inflammatory cytokines and growth factors [26]. Many studies have been published regarding humoral factors in PRPs and their effects [27,28,29], and we have also previously reported that the concentrations of humoral factors differ between PRPs derived from healthy subjects and OAK patients [27]. However, only a few studies have reported on the effect of PRPs on macrophages and their polarizations.

Macrophages are present in all tissues, play an important role in innate immunity, and are essential for early tissue repair of damaged or inflamed areas [30,31]. Macrophages respond to IP-10, MCP-1, CXCL9, and CX3CL1, infiltrate damaged or inflamed areas, and polarize into two functionally different types (M1 and M2) depending on their microenvironment. M1 macrophages are stimulated by IFN-γ, TNF-α, and LPS and release pro-inflammatory factors such as IL-1β and TNF-α. M2 macrophages are stimulated by IL-4 and IL-13 and release anti-inflammatory factors such as IL-10 and growth factors such as TGF-β, which are known to suppress inflammation and induce tissue repair [32,33,34]. Inflammation occurs in most synovium of OAK patients, and pathology progresses with an imbalance of M1/M2 macrophages in the synovium and synovial fluid caused by an increase in M1 macrophages and a decrease in M2 macrophages [17,35,36].

Therefore, the purpose of this study was to compare in PRPs derived from two clinically relevant kits the concentrations of typical cytokines and growth factors associated with M1/M2 macrophages and verify the effect of PRP supernatants on macrophage polarization. We first investigated the blood components and humoral factors in LP-PRP and APS, which we have clinical experience administering to OAK patients [27]. We first confirmed that compared to whole blood, APS contained higher levels of leukocytes, while LP-PRP was leukocyte poor as expected. 

Analysis of humoral factors in LP-PRP and APS showed that each contained different concentrations of humoral factors, among which both M1 and M2 macrophage related factors were found to be at a higher concentration in APS. APS is prepared with a dehydration process using polyacrylamide beads, which is reported to result in higher concentrations of humoral factors [37]. In fact, APS contained a higher concentration of M2 macrophage-related factors such as IL-10, an anti-inflammatory factor, and TGF-β, a growth factor. At the same time, M1 macrophage-related factors such as TNF-α, a pro-inflammatory factor, were contained at a higher concentration in APS than in LP-PRP. However, previous reports have shown that the effects of pro-inflammatory factors are negated by the higher concentrations of anti-inflammatory factors [38]. Moreover, an explanation for the fact that the concentrations of TGF-β and IL-1RA were negligible for LP-PRP is that platelets were not activated by external factors prior to quantitative analysis. 

To investigate the effect of PRPs on M1/M2 macrophage polarization, PRP supernatants were added to the culture media of monocyte-derived macrophages. Specifically, blood components were removed through centrifugation to prevent addition of monocytes derived from PRPs and to remove the effect of leukocytes contained in PRPs. The addition of PRP supernatants to macrophages resulted in the reduction of the expression of M1 macrophage markers. Furthermore, when PRP supernatants were added to the culture media of macrophages, they suppressed the M1 polarization of monocyte-derived macrophages (Figure 3B, Figure 4B) and promoted the polarization of monocyte–derived M1 macrophages to M2 macrophages (Figure 5). The effect of PRP supernatants on the macrophage phenotype observed in this study (Figure 4B,C) suggest that PRPs have little effect on M1 macrophage related factors that may polarize macrophages to M1 macrophages. These results indicate that PRPs may improve symptoms in OAK patients by polarizing M1 macrophages in joints to M2 macrophages.

In addition, reports have suggested that M2 macrophages can be categorized into three subsets: M2a macrophages induced by IL-4 and IL-13, expressing MRC1 and IL-10; M2b induced by signals from the immune complex, expressing IL-10 and major histocompatibility complex class II; and M2c induced by IL-10 and glucocorticoids, expressing MRC1, IL-10, and TGF-β [32,33,34]. Our results suggest that LP-PRP promotes the polarization to M2c macrophages and that APS specifically promotes the polarization to M2a macrophages. M2a macrophages are mainly related to anti-inflammatory activity while M2c macrophages to tissue repair [14,39]. As such, the mechanism of action of LP-PRP and APS may differ in the elimination of the imbalance of M1/M2 macrophages in the joint. 

PRPs have also been shown to enhance macrophage infiltration into tissues in tendon repair [20]. Furthermore, it is possible that monocytes contained in APS itself may differentiate and polarize to M2 macrophages once administered to the joint. Taken together, administration of PRPs to the knee joint of OAK patients may help eliminate the imbalance of M1/M2 macrophages in several ways: polarizing M1 macrophages already present in the joints to M2 macrophages; populating the joint with macrophages from the surrounding area and polarizing them to M2 macrophages; and populating the joint with monocytes contained PRPs and polarizing them to M2 macrophages.

This study has some limitations. First, because of the difficulty in recruitment, PRPs and monocytes used in this study were derived from peripheral blood of healthy subjects and not OAK patients. Second, we removed cellular components through centrifugation in experiments involving the addition of PRP supernatants. In addition to cytokines and growth factors, APS contains high concentrations of leukocytes, which may affect macrophage polarization, and their effect when administered to the joint must be further elucidated. For example, TNF-R released from leukocytes has been reported to inhibit the polarization to M1 macrophages [37]. Third, 60 mL of peripheral blood provide about 6 mL of LP-PRP or 2.5 mL of APS. Thus, the clinically relevant amount of PRPs used for intra-articular injection would result in different doses whereas equal amounts of LP-PRP or APS supernatants were added to the media in the macrophage experiments. Fourth, in standard treatment, PRPs are used immediately after purification whereas here they were frozen once.

This study is the first report on the effect of clinically relevant PRPs on macrophages and macrophage phenotypes. Our findings suggest that PRPs may help improve symptoms via modulation of macrophages in the joint, and warrant a further investigation of their effect on macrophages as a possible mechanism of action by which PRPs promote the tissue repair process. 

## 4. Materials and Methods

### 4.1. Ethics Statement

This study was reviewed and approved by the Institutional Review Board of the Tokai University School of Medicine (18R-134) and was conducted in compliance with relevant guidelines. Written informed consent was obtained from all participants.

### 4.2. PRP Purification

To purify PRPs, 120 mL of peripheral blood collected from each of the 12 healthy subjects (M = 5, F = 7, Age = 38.6 ± 11.0 years) was added to 12 mL of anticoagulant citrate-dextrose solution A (ACD-A; TERUMO, Tokyo, Japan) using two 60 mL syringes. Excess peripheral blood was centrifuged at 2200× *g* for 10 min and the top plasma layer was collected and stored at −80 °C until use.

LP-PRP was purified using a Cellaid Serum Collection Set P type kit (JMS, Hiroshima, Japan). This kit consists of primary and secondary containers connected at the top by multiple tubes. Blood (20 mL) containing ACD-A was injected into the primary container and centrifuged at 200× *g* for 15 min. The plasma layer containing platelets was transferred to the secondary container via a tube at the top and centrifuged at 1200× *g* for 15 min. Excess plasma was returned to the primary container, the pelletized platelets were disrupted by tapping, and then 2 mL of LP-PRP was collected. Approximately 6 mL of LP-PRP was collected from 60 mL of peripheral blood using 3 kits. One hundred micro liters was used immediately after for hematological analysis.

APS was purified using an Autologous Protein Solution (APS) kit (Zimmer Biomet, Warsaw, IN, USA). This kit consists of two independent tubes (a GPS3 III system and an APS Separator). Blood (60 mL) containing ACD-A was injected into the cell separation tube (GPS III system) and centrifuged at 745× *g* for 15 min using a dedicated centrifuge (Zimmer Biomet) and 6 mL of the upper layer (PRP layer) was collected. This 6 mL was added to the APS Separator and centrifuged at 219× *g* for 2 min in the same centrifuge and approximately 2.5 mL of APS was collected. One hundred micro liters was used immediately after for hematological analysis. The collected PRPs were stored at −80 °C until use. 

### 4.3. Hematological Analysis

The leukocyte, erythrocyte, and platelet concentrations of whole-blood, LP-PRP, and APS samples and leukocyte compositions of whole-blood and APS samples were determined using an automated hematology analyzer (XT-1800i; Sysmex, Kobe, Japan) immediately after preparation. 

### 4.4. Analysis of Humoral Factors

The concentrations of humoral factors in plasma, LP-PRP, and APS were measured using a flow cytometry bead-based immunoassay (LEGENDplex™ Custom Human 13-plex panel, BioLegend, San Diego, CA, USA) according to the manufacturer’s protocol. Plasma, LP-PRP, and APS were centrifuged at 16139× *g* at 4 °C for 5 min to remove cellular components and the supernatants were analyzed without any external activation. GM-CSF, M-CSF, IFN-γ, IL-4, IL-10, IL-13, IL-1RA, free active TGF-β1, TNF-α, MCP-1, IP-10, CXCL9, CXCL10, and CX3CL1 were measured simultaneously. Data were acquired using a FACS Verse™ Flow Cytometer (BD Bioscience, San Diego, CA, USA) and analyzed using BioLegend’s cloud-based LEGENDplex™ Data Analysis Software.

### 4.5. Isolation of Monocytes

Peripheral blood mononuclear cells (PBMCs) were separated from the buffy-coat of six healthy donors (M = 3, F = 3, Age = 32.0 ± 1.7 years) using a density gradient (Histopaque 1077, Sigma-Aldrich, St. Louis, MO, USA). PBMCs were washed with wash buffer consisting of Dulbecco’s phosphate-buffered saline (DPBS; Gibco, Waltham, MA, USA) and 1% bovine serum albumin (BSA; Sigma-Aldrich) and centrifuged at 538× *g* at 4 °C for 5 min. Contaminating red blood cells were hemolyzed with red blood cell lysing buffer (Sigma-Aldrich) for 10 min at 37 °C. Wash buffer was added and the cell suspension was centrifuged at 538× *g* at 4 °C for 5 min. The cell pellet was disrupted, FcR blocking reagent (Miltenyi Biotech, Bergisch, Gladbach, Germany) was added, and the mixture was kept at room temperature for 15 min to inhibit Fc receptor-mediated nonspecific antibody binding. Cells were stained with mouse anti-human CD14-allophycocyanin (APC) (BD Bioscience) at 4 °C for 30 min. After washing with wash buffer, the anti-APC microbeads (Miltenyi Biotech) were bound to cells at 4 °C for 20 min. The cells were washed with wash buffer and the CD14 + monocytes were isolated using an autoMACS Pro Separator (Miltenyi Biotech). Collected cells were counted with a particle counter (Sysmex).

### 4.6. Cell Culture

A modified version of a previously reported protocol was used [40,41,42].

The cells were cultured in Roswell Park Memorial Institute (RPMI) 1640 media containing GlutaMAX^TM^ supplement (Gibco) supplemented with heat-inactivated 10% fetal bovine serum (AusGeneX, Molendinar, Australia) and 1% penicillin-streptomycin (FujiFilm, Tokyo, Japan) (hereinafter called basal medium).

#### 4.6.1. Preparation of Monocyte-Derived Macrophages and Addition of PRP Supernatants

A summary of the process is shown in Figure 3A. Isolated monocytes were seeded at a density of 1 × 10^5^ cells/cm^2^ on an Upcell Multi 24 well plate (CellSeed, Tokyo, Japan) containing basal medium supplemented with 20 ng/mL M-CSF (Peprotech, Rocky Hill, NJ, USA), and incubated at 37 °C, 5% CO_2_. After six days, the medium was replaced with medium with each of the following supplements: monocyte-derived macrophages (MDM control group)—basal medium; M1 polarized group—basal medium + 50 ng/mL IFN-γ (Peprotech) + 100 ng/mL lipopolysaccharides from *Escherichia coli* (LPS, Sigma-Aldrich); M2 polarized group—basal medium + 20 ng/mL IL-4 (Peprotech); LP-PRP-added group—basal medium + 10% LP-PRP; APS-added group—basal medium + 10% APS. PRPs were centrifuged at 16,139× *g* at 4 °C for 5 min to remove cellular components and the supernatants were used. The cells were cultured for another two days and then used for analysis. MDM control group: 6 monocyte donors, 5 experiments, total *n* = 30; LP-PRP- and APS-added groups: 6 monocyte donors, 12 PRP donors, *n* = 72 group; M1 and M2 polarized groups: 6 monocyte donors, 2 or 3 experiments, *n* = 14 per group.

#### 4.6.2. Preparation of M1 Macrophages and Addition of PRP Supernatants 

A summary of the process is shown in Figure 5A. Isolated monocytes were seeded at a density of 1 × 10^5^ cells/cm^2^ on a 96 well plate (Thermo Fisher Scientific, Tokyo, Japan) with basal medium supplemented with 20 ng/mL M-CSF and incubated at 37 °C, 5% CO_2_.

After six days, the medium was removed. Basal medium was added to MDM control group, and basal medium + 20 ng/mL IL-4 was added to M2 polarized group. Basal medium + 50 ng/mL IFN-γ + 100 ng/mL LPS was added to the other groups. All cells were cultured for two days, and then the medium was replaced by the following: MDM control, M2 polarized, and M1polarized groups—basal medium; M1-M2 polarized group—basal medium + 20 ng/mL IL-4; LP-PRP-added group—basal medium + 10% LP-PRP; and APS-added group—basal medium + 10% APS. PRPs were centrifuged at 16,139× *g* at 4 °C for 5 min to remove cellular components and the supernatants were used. After two days, the medium was removed, the cells were washed with DPBS, and the total RNA was collected using Isogen II reagent (Nippon Gene, Tokyo, Japan). MDM control, M1 polarized, M1-M2 polarized, M2 polarized groups: 6 monocyte donors, 1 experiment, *n* = 6 per group; LP-PRP- and APS-added groups: 6 monocyte donors, 12 PRP donors, *n* = 72 per group.

### 4.7. Flow Cytometry

To collect cells, FACS buffer (DPBS + 1% BSA) was added to the Upcell Multi 24 well plate (method 4.5.1). The plates were kept at room temperature for 30 min to promote detachment of the cells and the cells were collected by pipetting the contents of each well. FcR blocking reagent was added to the cell suspension and the cell suspension was kept at room temperature for 15 min to inhibit Fc receptor-mediated nonspecific antibody binding. Each cell suspension was divided into two tubes. In one tube, the cells were mixed with the following six mouse monoclonal anti-human antibodies and the cell suspension was kept at 4 °C for 30 min for multiple staining: CD80-PE (Clone: L307.4), CD86-BUV395 (Clone: 2331), CD163-FITC (Clone: GHI/61), CD206-BV421 (Clone: 19.2), CD14-APC (Clone: M5E2), CD45-BV605 (Clone: HI30) (BD Bioscience). Cells in the other tube were mixed with nonspecific fluorescent mouse IgGs as a negative control. The cells were reacted at 4 °C for 30 min and then washed with FACS buffer. Data on the stained cells were acquired using a BD LSR Fortessa ™ Flow Cytometer (BD Bioscience) and analyzed using FlowJo (Tree Star, Ashland, OR, USA).

### 4.8. Gene Expression Analysis

Cells were lysed using Isogen II reagent for RNA extraction and stored at −80 °C until use. The lysate was then thawed at room temperature, and 40% (final volume) deionized water was added. The sample was vortexed and centrifuged at 16,139× *g* and 4 °C for 15 min, 75% of the supernatant was transferred to a new tube, and an equal amount of 70% ethanol was added and then vortexed. Total RNA was extracted using an RNeasy Micro kit (QIAGEN, Valencia, CA, USA) according to the manufacturer’s protocol. The total RNA quantity and quality were determined using a NanoDrop Lite (Thermo Fisher Scientific). cDNA was synthesized from RNA (40 ng) using a QuantiTect reverse transcription kit (QIAGEN) and a Thermal Cycler GeneAmp PCR System 9700 (Thermo Fisher Scientific). cDNA (5 ng) was pre-amplified using TaqMan PreAmp Master Mix (Applied Biosystems, Waltham, MA, USA). For pre-amplification, the reaction volume was adjusted to 10 μL according to the manufacturer’s instructions. Using the Thermal Cycler GeneAmp PCR System 9700, the DNA was denatured at 95 °C for 10 min, and then at 95 °C for 15 s and 60 °C for 4 min for 14 cycles. The amplified product was diluted 20-fold with TE buffer (1 ×) and used for qRT-PCR. qRT-PCR was performed using TaqMan fast advanced master mix (Applied Biosystems) with a QuantStudio 3 Real-Time PCR System (Applied Biosystems) at 50 °C for 2 min, 95 °C for 2 min, and then 95 °C for 1 s and 60 °C for 20 s for 40 cycles. The probes used for pre-amplification and qRT-PCR were GAPDH (Hs_02758991_g1), IL-1β (Hs_01555410_m1), IL-6 (Hs_00985639_m1), TNF-α (Hs_00174128_m1), MRC1 (Hs_00267207_m1), IL-10 (Hs_00961622_m1), and TGF-β (Hs_00998133_m1). The value of each gene expression (-ΔΔCt value) was obtained against the Ct of the internal control GAPDH and normalized to the control sample.

### 4.9. Statistical Analysis

Numerical results were statistically analyzed using SPSS^®^ Statistics Software 26 (IBM, Armonk, NY, USA). The data obtained was tested for normality of distribution through the Kolomogorov–Smirnov test and was rejected. Thus, the Wilcoxon signed-rank test was used for two-group comparisons, and Friedman’s test was used to compare three or more groups. The significance level was set at *p* < 0.05.

## 5. Conclusions

PRPs purified using two clinically relevant kits contained different concentrations of humoral factors, among which both M1 and M2 macrophage-related factors were contained at a higher concentration in APS. The addition of PRP supernatants to the culture media of monocytes and M1 macrophages promoted their polarization to M2 macrophages, possibly in different ways. These results warrant a further investigation of the possibility that PRPs may act on M1 macrophages in the joint and synovium to improve OAK symptoms.

## Data Availability

The data presented in this study are available on request from the corresponding author. The data are not publicly available because of confidentiality issues.

## Abbreviations

APS	Autologous protein solution
CX3CL1	C-XXX-C motif chemokine ligand 1
CXCL9	C-X-C motif chemokine ligand 9
GAPDH	Glyceraldehyde 3-phosphate dehydrogenase
GM-CSF	Granulocyte macrophage colony-stimulating factor
IFN-γ	Interferon-gamma
IL-10	Interleukin-10
IL-13	Interleukin-13
IL-1β	Interleukin-1 beta
IL-1RA	Interleukin-1 receptor antagonist
IL-4	Interleukin-4
IL-6	Interleukin-6
IP-10	Interferon gamma-induced protein-10
LP-PRP	Leukocyte-poor platelet rich plasma
LR-PRP	Leukocyte-rich platelet rich plasma
MCP-1	Macrophage chemoattractant protein-1
M-CSF	Macrophage colony-stimulating factor
MDM	Monocyte derived macrophage
MRC1	Mannose receptor 1
OAK	Osteoarthritis of the knee
PRP	Platelet rich plasma
TGF-β	Transforming growth factor beta
TNF-α	Tumor necrosis factor-alpha
